# Do COVID-19 and SARS Gene Complexities and Variations Help Overcome the Knowledge Gap?

**DOI:** 10.7759/cureus.8439

**Published:** 2020-06-04

**Authors:** Mehwish Javed, Faheem Javed, Huseyin Ekin Ergin, Tun Zan Maung, Safeera Khan

**Affiliations:** 1 Internal Medicine, California Institute of Behavioral Neurosciences and Psychology, Fairfield, USA; 2 Anaesthesia, California Institute of Behavioral Neurosciences and Psychology, Fairfield, USA; 3 Medicine, California Institute of Behavioral Neurosciences and Psychology, Fairfield, USA

**Keywords:** sars-cov-2, sars, similarities, rna virus, epidemics, transmission, control, spread, symptoms, immune response

## Abstract

A whole new pathogen, to which humans have virtually no pre-existing immunity, has caused fear all over the world. Severe acute respiratory syndrome coronavirus (SARS CoV-2) is one of the types of human novel-coronavirus of the family coronavirus. The nature of transmission of the virus makes it one of the most infectious pathogenic diseases that has ever existed. Though the human coronaviruses have existed since the discovery of the human coronavirus 229E (HCoV-229E) and human coronavirus OC43 (HCoV-OC43) in 1960, it has been a challenge to develop an effective cure as well as vaccine for the diseases associated with coronaviruses. Commonly, human coronaviruses cause illnesses such as intestinal and respiratory tract illnesses. Nevertheless, the symptoms reflected after infection from the coronaviruses take some time before being identified. Thus, viruses can replicate and cause more harm to the human body before being detected. Moreover, research continues to explain why some gene variations in some individuals increase the risk of some infectious diseases, while others are not affected. Looking at gene variations in people infected with Coronavirus Disease 2019 (COVID-19) and studying how genes influence people's response to infection will help to develop a vaccine that will help strengthen the immune system. Knowing how the human genes respond to the virus COVID-19 will help to cure people more effectively.

## Introduction and background

Some viral diseases have caused fatal havoc on the human species since they are associated with high mortality rates as well as low prevention and treatment approaches. Currently, there is a high risk of the spread of the COVID-19 disease caused by the SARS CoV-2 viruses. SARS CoV-2 virus is one of the types of human novel-coronavirus of the family coronavirus. The nature of transmission of the virus makes it one of the most infectious pathogenic diseases that has ever existed. Though the human coronaviruses have existed since the discovery of the human coronavirus 229E (HCoV-229E) and human coronavirus OC43 (HCoV-OC43) in 1960, it has been a challenge to develop an effective cure as well as vaccine for the diseases associated with coronaviruses. Later in 2003, a coronavirus pandemic erupted in Guangdong, a Chinese province famous for live wild animal trade, and was identified as the severe acute respiratory syndrome coronavirus (SARS-CoV). SARS-CoV pandemic affected at least 37 nations causing 1879 deaths across the globe by 2005 when it was effectively controlled [1]. Commonly, human coronaviruses cause illnesses such as intestinal and respiratory tract illnesses. Nevertheless, the symptoms reflected after infection from the coronaviruses take some time before being identified; thus, the viruses can replicate and cause more harm to the human body before being detected. Antibiotic treatment has been ineffective in treating infections due to coronaviruses, which acts as another factor contributing to the very high death toll [1]. Through conducting this research, this paper will establish reliable information on the transmission mode of coronaviruses and explore the various traits of both SARS-CoV and SARS-CoV-2 while offering various measures to control the implications of the viruses on the human population. Older people with pre-existing diseases and men are more susceptible to this infection and make up the majority of the victims. However, most people with severe symptoms are middle-aged adults who were stable at an early stage. Several studies conducted to determine the cause of such variation by collecting data from around the world, in particular from countries with a large number of cases, to find clues to help find a common denominator that can help in finding a cure. Age, sexual orientation, socio-economic status, and access to the health care system are some of the variants that were not very helpful, but researching genome could help answer the question we were looking for, a cure to combat this deadly viral infection and avoid another wave of similar diseases.

## Review

We used PubMed as our main database to search for the relevant published literature. We searched for all types of the articles that were related to the topic.

Animal to human transmission

The infections caused by the coronaviruses family to human beings since the onset of the 21st century influenced researches to identify the origin and transmission mode of the viruses across human beings. A study conducted to identify the initial cause of the severe acute respiratory syndrome disease revealed that the SARS-CoV virus was transmitted from the wild animal reservoirs in the Guangdong animal market. The direct or indirect interaction between human beings and animals, such as bats, was identified as the critical source of the coronavirus. Direct consumption of bats or being close to them, facilitated the spread of the virus from the wild animals to human beings, thus increasing the impacts of the disease onto the human population [2,3]. Similarly, the COVID-19 disease caused by SARS CoV-2 coronaviruses originally erupted from the interaction of human beings and wild animals such as bats in the Wuhan market in the Hubei province of China. Coronaviruses have been identified in various wild animals, especially mammals hence, illustrating their potential impacts on the human species. Though the viruses do not have a severe effect on the wild animals, they have been identified to influence severe respiratory infections that are resistant to known antibiotics as well as treatments.

Human to human transmission

The contact between infected persons and uninfected persons influences the spread of both the SARS CoV-2 and COVID-19, making them one of the most infectious diseases on the globe. Since both infections affect the respiratory tract, the viruses can spread via the cough droplets, sputum, and closeness, such as touching an infected individual. Moreover, the SARS CoV-2 viruses were discovered in the feces of a child infected by the disease, thus, depicting that fecal contact could facilitate the spread of the disease [4]. Further, the diseases influence the human species respiratory issues such as influenza, thus possible to spread via the air from one individual to another. COVID-19 has been identified as a disease affecting the upper respiratory tract during the first few days after an infection where it causes pain in the throat and later moves down to the lower respiratory tract. Disposing of the viruses on the skin after circulating across the body system increases the possibility of spreading the viruses via sharing clothes, body contact, shaking hands, and hugging [5]. Crowding and high socialization among human beings increase the chances of infections across the human population; hence, people need to avoid such situations.

Incubation period

The incubation period of a disease is the duration that the symptoms take to first appear after exposure to infection. A long incubation period depicts that an individual has a high tendency to infect other individuals before the disease can be medically diagnosed. During the incubation period, the pathogens causing the disease replicate, and the host immune response is activated producing cytokines such as interferon that can have global effects, hence, reducing the body resistance posed by the immune system [6]. Although an incubation period might be longer such as 14 to 21 days after exposure, the ability to infect other individuals can be before the symptoms are identified. Moreover, determining the length of the incubation period of certain infectious diseases provides the most probable time frame, during which an individual can be subjected to isolation and intensive medical observation [6]. The outbreak of severe acute respiratory syndrome in 2003 caused by the novel coronaviruses influenced studies on the coronavirus family and its traits. During the study, the incubation period for the disease was estimated to be a mean of 6.4 days. Currently, the estimated incubation period for the prevalent COVID-19 has faced various changes since different people are affected differently by the disease. According to the World Health Organization, the incubation period of COVID-19 is between two to 10 days [7]. The China National Health Commission had initially approximated the COVID-19 incubation 10 to 14 days.

On the other hand, the Centers for Disease Control and Prevention (CDC) approximates that the incubation period of the COVID-19 is two to 14 days [7]. Quarantine, self-isolation, and intensive medical examination are carried out to prevent the spread of the COVID-19 in public. Since there is no known cure or vaccine for the current global pandemic, many nations have opted to control the spread of the disease across the population to avoid fatal impacts such as high mortality rates.

Genomic differences between SARS-COV and SARS-CoV-2 viruses

Genomic traits of disease-causing pathogens reflect their origin as well as establish their implications on the host body. Further, the study of the genomic characteristics of a disease-causing agent influences the development of possible treatment measures that can be used to address the effects of the infection across the affected population [8]. Current researches conducted on nine patients depicted that the human novel coronavirus 2019 has a ten genomic sequence similar to two bat novel coronavirus-like pathogens with a similarity of 99.98% across the examined nine patients [8,9]. The bat-SL-CoVZ45 and bat-SL-CoVZXC21 are distinct from the SARS-CoV virus that affected at least 37 nations in 2003 [9]. Studies have shown that despite some variations in genomic sequences, COVID-19 has a similar receptor-binding domain structure to that of SARS-CoV. Moreover, the 2019 SARS-CoV-2 is categorized as a subgenus Sarbecovirus of the genus Betacoronavirus [8,10]. Additionally, the close relation of the SARS-CoV-2 infection with the bat-like coronaviruses shows that close contact between humans and bats could have influenced the spread of the virus into the human hosts. Moreover, the mutation of the SARS-CoV is long term since it can take years; hence the resistivity to vaccines could be low but develop within several years of exposure [11]. Currently, the mutation rate of the SARS-CoV-2 virus is not known, though its replication rate is high and poses fatal challenges to human health.

Immune response and gene variations

The gene, known as human leukocyte antigen (HLA), produces proteins that bind to the pathogen that acts as warning signs to alert immune cells. The human body has three different classes of proteins to determine how the body can best fight certain infections, including SARS-CoV-2. If HLA binds a viral protein that is critical for the pathogen to replicate and survive, the subsequent antibody activity is likely to be more effective in targeting the virus. If someone was previously exposed to common coronaviruses - which cause the common cold - and had the right HLA types that provide some protection against other viruses, it is theoretically possible that they could also generate an early immune response against the novel SARS-CoV-2 virus [12].

Immune system response is different in different people. Overreaction of the immune system, which is known as cytokine storm, is thought to be the leading cause of death in many severe acute respiratory syndrome (SARS) patients and is believed to be responsible for some of the deaths among young COVID-19 patients. The mortality rate among men infected with COVID-19 is higher than mortality rate among women with COVID-19, partly due to angiotensin-converting enzyme 2 (ACE2) found in the X chromosome, which means that men have one of the genes that make them more vulnerable to this disease. In comparison, women have two copies of the gene; ACE2 would mitigate any viral susceptibility to one variant from the second allele of ACE2 [13].

To date, some genetic studies suggest that variants in HLA genes are likely to play a role, while others point to the difference in ABO blood type and ACE2 genes that SARS-COV 2 binds to human cells. In addition to the genetic variants of the ACE2 receptor, the severity of the disease is influenced by variations in human leukocyte antigen genes that affect the immune response to the virus.

Wuhan University research tested different sources of ABO blood from 369 virus-infected patients and found 32.16%, 24.90%, 9.10%, and 33.84% for A, B, AB, and O, respectively. It has proposed a high type A contingent and a low type O contingent. These results reflected blood tests in another hospital, followed by 37.75%, 26.42%, 10.03%, and 25.80%. A similar pattern has been reported for mortality rates, with 206 deaths at 41.26%, 24.27%, 9.22%, and 25.24%, respectively in classes A, B, AB, and O [14]. Based on these findings, it is suggested that people with blood type "A" are at significantly higher risk of infection than people with other ABO blood-types, which may be due to the presence of anti-A antibody in the blood. However, more clinical trials are needed to support this association of ABO blood groups and COVID-19.

The association between the levels of ACE2 expression and the four key levels of immune signature in the tissues, including CD8+T cells, interferon response, B cells, and natural killer (NK) cells, was evaluated. According to the human protein atlas (HPA), significantly higher levels of ACE2 protein and gene expression are found in the gastrointestinal tract, kidney, gallbladder, and male reproductive tissues. This data could also explain the higher number of cases of COVID-19 in males compared to females [15].

Looking at the interaction of ACE2 levels of expression with CD8+T cells, interferon, B cells, NK cells in males, there was a substantially positive linkage in the thyroid, heart, adrenal glands, liver, kidneys, colon, and bladder. Whereas, the females only had interaction between ACE2 and immune markers in thyroid, heart, and colon, which indicates the variations and similarities between male and female expressions of ACE2 and immune markers. It also explains why some people have respiratory symptoms, and others have gastrointestinal symptoms such as diarrhea, nausea, vomiting, and other signs of the system. It has already shown that the lung cells are more easily contaminated with SARS 2 due to higher levels of ACE2. Male and older age groups have a higher degree of association between ACE2 expression and immune signature than females and young adults, which explains the higher level of SARS 2 cases and death in this population. There are no significant differences in ACE2 expression between males and females, but the distinction lies in the immune response that varies from person to person [15].

Diagnosis for both viruses

The diagnosis of coronaviruses is based on the symptoms reflected by the affected individual. Since both the SARS-CoV and SARS-CoV-2 cause a severe influenza-like infection that affects the human respiratory system, respiratory symptoms are commonly used to detect the viruses [16]. The initial indications of the presence of the virus in the human body include coughing, running nose, common cold, headaches, body aches, and difficulty in breathing. Further, various more advanced techniques are used to determine the type of the virus as well as its genetic composition in the human respiratory system [17]. Electron microscopy, reverse transcriptase-polymerase chain reaction (RT-PCR) amplification, serology, and sequence-independent amplification are the fundamental techniques used to develop the essential traits of both SARS-CoV and SARS-CoV-2 in the human body [M]. Currently, RT-PCR amplification, serology, and sequence-independent amplification are commonly used approaches to diagnose the SARS-CoV-2 virus across the human population.

Treatment measures

The treatment process of the SARS-CoV infection varies slightly from the methods currently employed to address the increasing issues of SARS-CoV-2 disease on the globe. SARS-CoV treatment measures involved the initiation of ventilators that assisted the patients in accessing effective breathing ability [18]. Treatment of the subsequent infections facilitated by the virus enhanced the ability to control the disease though it eventually caused at least 1800 deaths [19]. On the other hand, the current treatment measures imposed to counter the fatality of the SARS-CoV-2 include the treatment of the conditions associated with the infection since there is no known cure for the disease. Further, scientists are considering the application of virus antiviral medication to assist in curbing the prevalence of the disease across the human population. The close association of SARS-CoV and SARS-CoV-2 has influenced the use of ventilators to enhance the breathing ability of patients affected by SARS-CoV-2 [20]. Controlling the impact of SARS-CoV-2 has been a problematic issue across the globe because of its high infectious condition and its generic composition. The protein coat over the key genetic ribonucleic acid (RNA) material inside the SARS-CoV-2 virus resists the impacts of any treatment chemical utilized to cure the ailment [21]. Currently, regular washing of hands, avoiding social contact with an infected individual as well as wearing protective masks and gloves are the commonly used measures to prevent the spread of the coronaviruses across the human population. Isolation and quarantine measures are being imposed on the individuals who have tested positive with the disease.

Plasma transfusion

Convalescent plasma is used as a clinical procedure and as an option for patients who are either seriously ill or who do not respond to other therapies. This plasma comes from patients who have recovered from COVID-19 infection and who have developed antibodies to control the virus. These antibodies are formed in plasma to protect against possible infections with the same virus. However, the potential treatment benefits and risk of developing side-effects need to be further investigated. The use of convalescent plasma in previous Middle East Respiratory Syndrome (MERS-CoV) and SARS outbreaks demonstrated safety and faster viral clearance, particularly when given early in the course of the disease. Transferring plasma from patients who developed neutralizing antibodies (nAbs) to specific SARS-CoV-2 proteins could neutralize the virus, prevent further replication and stop ongoing tissue damage in patients with less severe infections, earlier in the course of the disease, or prophylactically administered to high-risk individuals [22].

Understanding the actions of crucial COVID-19 proteins is critical to the development of plausible treatment or vaccines. CoV-2 S protein plays a vital role in the fusion, infection, and transmission of viruses. Moreover, the most likely objective is to build nAbs to block the binding and merging of COVID-19. The S protein is the primary inducer of nAbs, consisting of two components, S1 and S2. The receptor-binding domain (RBD) interacts directly with the host receptor S1. Although SARS 2 uses the same ACE2 receptor as SARS for cell entry, SARS-CoV-2 S protein binds ACE2 with a higher affinity than SARS-CoV, suggesting that its ACE2 recognition may differ from SARS-CoV. Besides, there is a minimal cross-reactivity of the antibody between the two virus S proteins. Some studies indicate that SARS-CoV antibodies may be binding to SARS CoV-2 RBD, but their ability to neutralize is not understood.

SARS and MERS unique nAbs that include monoclonal antibodies (mAbs) target regions S1 and S2 and block RBDs to their respective receptors and interfere with S2-mediated membrane fusion or host cell entry, preventing viral infection. Despite the use of polyclonal antibodies to treat SARS 2 disease in recovered COVID-19 patients, researchers have yet to discover novel neutralizing mAbs for SARS-CoV 2. If these antibodies developed, it will take several years for them to be ready for human use, as their safety and efficacy should be tested [23].

Data from various human and animal-acquired viral infections and vaccines indicate that neutralizing antibodies (nAbs) are needed to prevent or cure viral infections. These nAbs may be either contaminated or vaccinated to avoid viral infections. Lack of data on the role of nAbs in disease progression due to possible heterogeneity of SARS 2-specific nAbs in recovered COVID-19 patients needs further investigation in the development of an appropriate vaccine and patients undergoing convalescent plasma therapy.

In the past, antibody therapy for plasma infusions has been commonly used to treat viral infections, including SARS and MERS. The efficacy of this therapy has been linked to the concentration of nAbs in the recovered plasma donor. If viral infection with SARS-CoV-2 persists, convalescent plasma continues in recovered patients or serum is used as an alternative therapy.

nAb levels in patients recovered from COVID-19 may vary, some with high nAb titers, some with very low titers, indicating that other immune responses, including T cells or cytokines, may contribute to the recovery of these patients. Research has shown that higher C-reactive protein (CRP) and lower lymphocyte counts have a strong innate immune response at the time of admission to elderly patients than younger patients, which may have caused an adverse condition following coronavirus infection. Rapid innate immune responses in older patients often suggest higher rates of nAb titer. It attributes to the fact that neutralizing antibodies (nAbs) have a positive association with CRP but are negatively associated with the indication of patient lymphocytes, a link between cell-mediated antibody response and immune response [24].

Comparative analysis

The impacts of coronaviruses on the globe have caused fatal situations that led to the death of more than 25,000 individuals globally up to this day. SARS-CoV-2 is a very highly infectious disease that can spread via close contact between human beings [25]. Coming into contact with surfaces that contain the virus further facilitates the spread of the virus. Since humans are social beings participating in social events, it increases the risk of spreading the virus from one individual to another. The traits of the virus make it more likely to affect a large number of individuals across the globe. Moreover, the SARS-CoV-2 disease is a viral disease caused by a novel human coronavirus that has a long strand of RNA, hence, it is complex to regulate its replication in the human body [26]. COVID-19 viruses have protein spikes that enhance their compatibility with the human cells; therefore, they can survive in the human body.

The coronaviruses multiply at a higher rate; hence, they spread through the affected human organs at a high rate. The high replication rate increases the rate by which the viruses spread across the human body, hence it is challenging to cure the disease. The compatibility of the viral cells with human cells further inhibits treatment approaches aimed at denaturing the viral cell. The key difference between the SARS-CoV virus and the SARS-CoV-2 virus is their genomic structure, and their response to different treatment approaches. SARS-CoV genomic make-up varies from the SARS-CoV-2 since the SARS-CoV-2 virus has longer RNA strand than the strand in the SARS virus. The RNA S-sequence in both SARS-CoV-2 and SARS-CoV viruses is similar; hence, the key differences have not been effectively proven [27]. The impacts of the diseases on human bodies are identical though the SARS-CoV affects various organs.

Currently, there are different facts about the SARS-CoV-2 that are not known through current researches that are being conducted to answer the different concerns on the SARS-CoV-2 infection. The treatment approaches in terms of vaccine and a medicinal cure have not been discovered. The survival of the COVID-19 virus in the human corpse after demise is not currently known; thus, the researches conducted are aimed at informing the relevant risk eminent when one comes into contact with the corpse of corona patient [28]. People are encouraged to maintain a social distance to avoid further spread of the disease. Moreover, frequent hand-washing and disinfecting the possibly contamination-prone surfaces is recommended. Using face masks and gloves prevents individuals from the virus from the surfaces as well as from the other infected individuals. Staying at home has been advocated across the globe, where individuals are encouraged to conduct essential activities from their homes and avoid moving out of their houses for unnecessary reasons. Comparison between SARS and COVID-19 is shown in Table 1.

**Table 1:** The differences and similarities between SARS and SARS COV-2

Features Comparison	SARS	SAR-COV-2
The first case diagnosed	November 2002 Guangdong, China	December 2019 Wuhan, China
Mode of transmission	The animal market mainly from bats to human	Animal source- pangolins or bats to human
Spread/deaths worldwide	>8000 cases were reported worldwide, with almost 800 deaths	4,311,914 confirmed cases globally, with 290,597 deaths as of April 25, 2020
Virus	RNA	RNA
Incubation period	2-14 days	2 to 14 days
Diagnostic test	RT-PCR, rRT-PCR, RT-LAMP, rRT-LAMP, swabs, lower respiratory tract (LRT) aspirate or bronchoalveolar lavage	RT-PCR, CXR, CT chest, Swabs, LRT aspirate or bronchoalveolar lavage
Treatment	No specific treatment or vaccine available	No specific treatment or vaccine available

SARS and COVID-19 have similarities, but can they co-infect a patient? If they do co-infect, then how often do they have a synergistic effect, if they do have a synergistic effect, then is there a way to differentiate them from each other symptomatically? Can they both be differentiated based on signs and symptoms when they co-infect a patient?

Symptom wise COVID-19 symptoms vary. Why these variations exist? Why some patients exhibit some symptoms while others exhibit different symptoms? Is this because of the variant of the virus? Or is it because of the genetic differences between the individuals? And the way COVID-19 manifests with different symptoms in different individuals, is it the same situation with SARS? If yes, then to what extent?

These are the questions that arise, and we recommend future scientists to explore more about these questions.

## Conclusions

We have much to learn from previous outbreaks of coronaviruses and the new ongoing pandemic, by looking at the similarities and differences between the current SARS-CoV-2 and the previous outbreak of SARS; a remarkable resemblance emerges with some unique features of its own. Every time a new strain of these viruses appears, it is more aggressive than the previous ones, which makes it more difficult to find treatment and a vaccine. A clear understanding of the host immune response may help in finding how the virus causes disease and a possible cure to eradicate this virus. The wet animal markets have played a significant role in the SARS, and COVID-19 global epidemics emphasize the need for a change in China - away from their use. To prevent zoonotic transmission, the best approach is to suspend or close down these markets; however, due to its long history in China, it will be impossible to shut down permanently as the income of most people relies on selling live animals. The best possible way to prevent and control the disease spread is the use of proper hygiene, and rules for limiting animal-human contact would be ideal, in addition to epidemiological surveillance and monitoring. The knowledge we have so far is limited, and more research work is needed to design prophylactic and therapeutic measures to fight current epidemics and any future outbreak of similar coronavirus.
